# Altered parietal multisensory integration in chronic tinnitus during closed-loop real-time fMRI auditory downregulation

**DOI:** 10.1016/j.nicl.2026.103960

**Published:** 2026-02-12

**Authors:** Nicolas Gninenko, Pascal Senn, Sven Haller, Dimitri Van De Ville

**Affiliations:** a Neuro-X Institute, Ecole Polytechnique Fédérale de Lausanne (EPFL), Campus Biotech, 1202 Geneva, Switzerland; b Department of Radiology and Medical Informatics, University of Geneva (UNIGE), 1211 Geneva, Switzerland; c Faculty of Science and Medicine, Department of Neurology, University of Fribourg, 1700 Fribourg, Switzerland; d Department of Otorhinolaryngology-Head and Neck Surgery, Geneva University Hospitals, 1211 Geneva, Switzerland; e Centre d’Imagerie Médicale de Cornavin (CIMC), 1201 Geneva, Switzerland; f Faculty of Medicine, University of Geneva (UNIGE), 1211 Geneva, Switzerland; g Department of Surgical Sciences, Radiology, Uppsala University, 751 85 Uppsala, Sweden

**Keywords:** Auditory cortex, Chronic subjective tinnitus, Downregulation, fMRI neurofeedback, Multisensory integration, Parietal operculum

## Abstract

Chronic subjective tinnitus is the most common form of tinnitus and refers to an internal persistent phantom auditory perception. Evidence from neuroimaging studies has established tinnitus and its associated distress as a brain network disorder, with multiple brain regions displaying dysregulated activity or connectivity extending way beyond the auditory pathway. Hyperactivity in the auditory cortex has been associated with perceived tinnitus loudness, and somatosensory-auditory interactions have recently been involved in promising treatment avenues through their disruption using non-invasive or bimodal neuromodulation techniques, albeit with low mechanistic evidence.

In this study, we evaluated the neural effects of prolonged downregulation of bilateral auditory cortex activity mediated by real-time fMRI neurofeedback in individuals with moderate to severe (Tinnitus Handicap Inventory [THI] scores ≥ 48) chronic tinnitus. Twenty-one participants (aged 49 ± 11.4 years old, 16 males, 5 females) completed 15 fMRI neurofeedback sessions over 3–4 months each, as part of a randomized clinical trial (ClinicalTrials.gov: NCT05737888) comparing neurofeedback interventions over the current standard of care, cognitive behavioral therapy (CBT). We performed whole-brain general linear modeling analyses to delineate regulated brain areas, accounting for age, gender, and THI scores at baseline. Task-modulated functional connectivity analyses were carried out using psychophysiological interactions to unveil associated connectivity patterns emerging during cognitively demanding tinnitus defocalization.

Most participants succeeded at reducing the average activity of their auditory cortex throughout the training. Whole brain analyses additionally revealed a strong downregulation of parietal operculum 3 (OP3), a region previously reported to be activated in the right hemisphere during experimentally induced transient phantom percepts. In accordance with the hypothesis that OP3 may mediate the integration of multisensory inputs in tinnitus, we have shown that both left and right auditory cortices decrease their connectivity with OP3 during closed-loop auditory downregulation. Moreover, we observed a reduced connectivity of bilateral OP3 with a functional multisensory integration network that was previously found to be engaged by the primary and secondary auditory cortices during audio-tactile integration.

These findings support the hypothesis of OP3 having a key role in multisensory integration stemming from altered somatosensory-auditory crosstalk in chronic tinnitus. Targeted neuromodulation to desynchronize the connectivity between OP3 and the auditory cortex could further inform our understanding of the mechanisms behind recent successful bimodal interventions for reducing tinnitus.

## 1 Introduction

Subjective tinnitus refers to an internal phantom auditory perception, usually in the form of a tone or noise, not triggered by any external sound source (Shore et al., 2016). It is a highly heterogeneous symptom (Cederroth et al., 2019, Beukes et al., 2021), which is estimated to affect around 15% of the general population, and is perceived as a major burden by over 120 million people worldwide, with an incidence rate slightly above 1000 per 100′000 person-years (Jarach et al., 2022). While some forms of acute tinnitus may resolve over time, chronic tinnitus — that is nowadays considered as such after 3 months (Ridder et al., 2021), — is often accompanied by anxiety (Pattyn et al., 2016), depression (Ziai et al., 2017, Salazar et al., 2019), insomnia and sleep disturbances (Gu et al., 2021, Weber et al., 2022), or cognitive impairments (Tegg-Quinn et al., 2016, Neff et al., 2021, Yang et al., 2024). Its associated distress has also been linked to certain personality traits, such as high neuroticism or high stress reaction (Durai and Searchfield, 2016). Subjective tinnitus differs from its rare objective counterpart, which is sometimes audible to an external examiner, and can be attributed to hyperactivity of outer hair cells resulting in abnormal otoacoustic emissions in the inner ear (Ohki and Kikuchi, 2013). Common causes of chronic tinnitus typically involve initial cochlear damage triggered by elevated noise exposure, sudden hearing loss, or adverse reactions to ototoxic drugs (Langguth et al., 2013). Progress in understanding the neuropathophysiology of tinnitus has been hindered by its tight association with other audiological disorders, such as hearing loss (Tan et al., 2013) or hyperacusis (Cederroth et al., 2020). Although cochlear implantation has demonstrated beneficial effects on tinnitus in specific cases with severe hearing loss (Assouly et al., 2021, Borges et al., 2021, Yuen et al., 2021), the latter is not recommended for tinnitus patients with near-normal audiograms. No evidence-based universal cure for tinnitus is currently available (McFerran et al., 2019), but several treatments have been attempted with varying degrees of success (Zenner et al., 2017). As of today, cognitive behavioral therapy (CBT) is still the most widely recommended treatment for chronic tinnitus (Zenner et al., 2017, Fuller et al., 2020). CBT aims at reducing the negative impact of tinnitus, in particular its induced distress and handicap (Cima et al., 2012), but not at directly influencing acoustic features of the percept itself, such as its loudness (Jun and Park, 2013).

The multitude of clinical endeavors aimed at eliminating tinnitus often fall short when faced with the heterogeneity of the symptom, especially since most interventions are aimed at alleviating tinnitus-associated distress (Ridder et al., 2021, Czornik et al., 2022), rather than the tinnitus percept itself. Thus, it is unlikely that a single tinnitus treatment will benefit a large group of individuals with different levels of tinnitus severity, despite early efforts to characterize its heterogeneity (Mohan et al., 2022, Guillard et al., 2023). The lack of effective treatments is partly due to an incomplete understanding of the neural mechanisms involved in tinnitus. Several pathophysiological models of tinnitus have been described (Langguth et al., 2024), ranging from peripheral auditory dysfunction to more centrally altered brain function (Eggermont and Roberts, 2004, Roberts et al., 2010), among others. While none of the models can comprehensively explain all the available clinical evidence on tinnitus, unifying representations based on the Bayesian mechanisms of predictive coding have been proposed (De Ridder et al., 2014, Sedley et al., 2016, Hullfish et al., 2019, Schilling et al., 2023), in which sensory precision plays an important role in dissociating potentially altered neural activity and tinnitus perception itself.

Accumulating evidence from recent neuroimaging research in tinnitus supports disruptions in brain regions extending beyond the sole auditory pathway (Elgoyhen et al., 2015), essentially advocating for tinnitus to be recognized as a brain network disorder (De Ridder et al., 2014, De Ridder et al., 2021), which entails that adaptive treatment strategies based on personalized biomarkers may hold promise for clinical benefit (Lan et al., 2022). Neuroimaging studies in tinnitus, particularly those using fMRI, have revealed the involvement of, and increased connectivity among, auditory and non-auditory brain areas, notably the amygdala, cingulate cortex, dorsolateral prefrontal cortex (dlPFC), insula, parahippocampus, and ventromedial prefrontal cortex (vmPFC) (Elgoyhen et al., 2015). Hyperactivity of the primary auditory cortex (A1) has also been associated with tinnitus loudness (Mühlnickel et al., 1998, Andersson et al., 2000, Weisz et al., 2007, Ortmann et al., 2011, Vanneste et al., 2011, Ghazaleh et al., 2017), although this relationship has been questioned (Geven et al., 2014). Furthermore, common pathophysiological mechanisms in tinnitus and chronic pain have also been discussed (De Ridder et al., 2011, De Ridder et al., 2021), with increased γ-band activity in both auditory and somatosensory cortices in both conditions (De Ridder et al., 2023), consistent with scarce evidence from intracranial recordings of the secondary auditory cortex in tinnitus (De Ridder et al., 2011). The involvement of a somatosensory pathway in tinnitus following acute acoustic trauma (AAT) has also been suggested (Job et al., 2012), with the idea that the emerging phantom percept may occur as a form of proprioceptive illusion due to the perpetuated mechanical damage to the middle ear after an acoustic shock, with a cortical representation in the parietal operculum 3 (OP3) (Job et al., 2016). The latter is presumably mediated by the trigeminal nerve (Shore et al., 2016, Shore, 2005), with functional connectivity (FC) findings supporting the view that tinnitus arising from AAT may require a permanent cognitive control to filter out the associated phantom percept (Job et al., 2020).

Non-invasive neuromodulation approaches such as repetitive transcranial magnetic stimulation (rTMS) (Londero et al., 2018, Liang et al., 2020) or (high-definition) transcranial direct current stimulation (tDCS) (Elyssa Kok et al., 2021, Jacquemin et al., 2021), among others, have gained growing interest for probing tinnitus-related changes with more spontaneous observable effects than those following therapies such as CBT. Similarly, protocols requiring the active cognitive involvement of participants in self-regulating presumed physiological correlates of tinnitus through EEG (Güntensperger et al., 2017) or fMRI neurofeedback (Haller et al., 2010, Emmert et al., 2017) have been investigated. EEG neurofeedback studies have attempted several upregulation (↑) or downregulation (↓) paradigms involving different frequency bands (e.g., α ↑ (Hartmann et al., 2013, Ma et al., 2022), α ↑ β ↓ (Gosepath et al., 2001, Schenk et al., 2005), α ↑ δ ↓ (Crocetti et al., 2011, Güntensperger et al., 2019, Güntensperger et al., 2020, Jensen et al., 2020), or even α ↑ β ↓ γ ↓ (Vanneste et al., 2018); see Güntensperger *et al*. for a detailed review). However, in addition to the diverse tinnitus subgroups involved, the variability in research protocols, electrode positioning, or source localization, as well as in the methods used for feedback computation and delivery, has led to a wide range of outcomes regarding long-lasting clinical efficacy of EEG neurofeedback. There is therefore a difficulty in reaching a consensus on a sound methodological approach in the field, even if initial efforts to identify individual success predictors with EEG neurofeedback have been made (Riha et al., 2021), as well as an investigation on the feasibility and efficacy of a portable solution ultimately brought to the patient’s home (Guillard et al., 2021).

In contrast, fMRI neurofeedback enables the assessment of spatially well-defined effects of auditory downregulation in the human brain. In Haller *et al*. (Haller et al., 2010), a small group of five out of six participants with chronic tinnitus successfully downregulated their broad auditory cortex activity over four short fMRI neurofeedback sessions, and two participants noted a slight improvement in their tinnitus after the training. Broad deactivations were found in bilateral A1, in the default mode network (DMN; prefrontal cortex, precuneus, and inferior parietal lobe), and increased activations in bilateral insula extending to the bilateral ventrolateral prefrontal cortex (vlPFC) and right dlPFC. In a follow-up methodological study (Emmert et al., 2017), continuously delivered feedback was found to be more advantageous than its intermittent alternative for prolonged fMRI neurofeedback training, and downregulation in parts of the secondary auditory cortex was found to be more pronounced than in A1. In the current study, we aimed to (i) investigate the neuronal correlates of auditory downregulation during prolonged fMRI neurofeedback in a group of patients with moderate-to-severe chronic tinnitus, in addition to the previously published positive clinical outcomes (Gninenko et al., 2024), and to (ii) identify patterns of task-modulated functional connectivity stemming from the bilateral auditory cortex during downregulation. Specifically, we hypothesized that such patterns may involve a key region in the parietal operculum (OP3), which likely mediates the multisensory integration of the tinnitus percept (Jaroszynski et al., 2022), in light of recent bimodal neuromodulation approaches targeting the auditory-somatosensory crosstalk in chronic tinnitus.

## 2 Materials and methods

This study presents neuroimaging findings from the fMRI group of the *NeuroTin* clinical trial (ClinicalTrials.gov: NCT05737888; Swiss ethics registration number: BASEC2017–00813). Clinical findings have previously been published (Gninenko et al., 2024). *NeuroTin* was a randomized, prospective clinical trial comparing the superiority of neurofeedback (fMRI and EEG) interventions over CBT, with the reduction of Tinnitus Handicap Inventory (THI) (Newman et al., 1996) scores as primary outcome. General functioning, sleep quality, state- and trait-anxiety, and depression, were assessed as secondary outcomes (Gninenko et al., 2024). The trial was funded by the Wyss Center for Bio and Neuroengineering (Geneva, Switzerland). The study protocol was approved by the local ethics committee and conducted in accordance with the principles of the Declaration of Helsinki.

### 2.1 Participants

Twenty-nine subjects with chronic subjective tinnitus were randomized into the fMRI neurofeedback group from Geneva University Hospitals between December 2017 and December 2021. Clinical characteristics of the cohort were described in a previous publication (Gninenko et al., 2024). In brief, patients with chronic (≥ 6 months), subjective, non-pulsatile, persistent, moderate to severe (THI ≥ 48) tinnitus, with functional hearing, were eligible for enrolment. Patients presenting contraindications to MRI, conductive hearing loss exceeding 20 dB at two or more frequencies, or any significant neurologic or psychiatric disease were excluded from participation. Participants were informed about the study procedure, and all provided written informed consent. No financial compensation was provided for fMRI neurofeedback, but all travel-related expenses to the investigation site (Campus Biotech, Geneva, Switzerland) were reimbursed.

### 2.2 Study design

#### 2.2.1 Procedure

Participants attended 15 weekly MRI visits spread over 3 to 4 months. Each visit consisted of six to seven fMRI neurofeedback runs of ∼6.5 min each. The number of runs per visit (six or seven) was defined together with each participant during the first visit, depending on perceived fatigue. Sometimes, up to two visits per week were scheduled to accommodate participants’ availability. The maximal time limit between two consecutive visits was set to 4 weeks. All MRI visits began with an anatomical T1 scan to obtain a high-resolution template for subsequent fMRI neurofeedback training. Functional localizers, resting-state fMRI (rs-fMRI), and deformation field map sequences were acquired prior to fMRI neurofeedback runs. Further details of the experimental procedure are summarized in the Supplementary Material, using the CRED-NF checklist (Ros et al., 2020).

#### 2.2.2 Data acquisition

MRI data was acquired on a 3 T Siemens MAGNETOM Prisma scanner with a 64-channel head and neck coil at Campus Biotech (Geneva, Switzerland). Anatomical imaging was performed using a T1 magnetization prepared rapid gradient echo (MPRAGE) sequence with a generalized autocalibrating partially parallel acquisition (GRAPPA, acceleration factor = 2) scheme, with repetition time (TR) = 2300 ms, anterior to posterior phase encoding (A ≫ P), echo time (TE) = 2.25 ms, inversion time (TI) = 900 ms, 208 × 256 × 256 resolution (x × y × z), flip angle (FA) = 8^◦^, isotropic voxel size (1.0 mm^3^), 208 volumes (∼5 min). Functional data (rs-fMRI [320 vol, ∼8 min], auditory localizers [230 vol, ∼5.5 min], and fMRI neurofeedback [270 vol. per run, ∼6.5 min per run]) were acquired using an interleaved multislice echo planar imaging (EPI) sequence (accel. factor = 4) with TR = 1500 ms, A ≫ P, TE = 31 ms, 108 × 108 × 64 with no gap, FA = 64^◦^, 2.0 mm^3^ isotropic. Acquisition parameters (in-plane resolution, number of z-slices, TR) were optimized during pilot recordings according to the data export and online processing available computing capabilities (Gninenko, 2022). Additional data acquisition details are available in the Supplementary Material.

#### 2.2.3 Functional localization of bilateral auditory cortex

A conventional auditory stimulation paradigm was used to delineate the fMRI neurofeedback target regions of interest (ROIs) (Haller et al., 2010, Emmert et al., 2017, Van De Ville et al., 2012, Haller et al., 2013). It was composed of a block-design 1 kHz sine tone amplitude-modulated at 6 Hz with 30 s ON, 30 s OFF (5 repetitions, starting with 30 s OFF), delivered binaurally through pneumatic earphones (MR Confon Starter f mkII+, Cambridge Research Systems, UK). This paradigm is known to elicit strong and lasting activation in the auditory cortex (Haller et al., 2006, Seifritz et al., 2002). Participants were instructed to remain still and focus on a white cross presented on a gray background on the MR-compatible screen. Individual bilateral auditory target ROIs were then manually created using SPM12 (fil.ion.ucl.ac.uk/spm, The Wellcome Centre for Human Neuroimaging, UK) in MATLAB R2016b (The MathWorks Inc., Natick, MA, USA) by inspecting activation contrasts with *p* < 0.05 (family-wise error corrected), or by selecting a minimal ROI size of at least 200 voxels. The same ROIs were retained throughout the 15 MRI visits for each participant.

#### 2.2.4 Functional MRI neurofeedback training and instructions to participants

During the first visit, participants were instructed to try to anticipate and develop at least one cognitive strategy to distract themselves from their tinnitus based on existing coping habits. This approach is rooted in the understanding that most individuals affected by chronic tinnitus have already developed coping and habituation strategies. Participants were encouraged to explore different cognitive strategies during the first few visits. By keeping a balance between an implicit fMRI neurofeedback paradigm (Sitaram et al., 2017) and individualized cognitive strategy guidance throughout the training, we hypothesized that such an approach could outperform a directed (i.e., explicit) training, by enabling participants to probe their own cognitive strategies and observe their effect on bilateral auditory deactivation (MacDuffie et al., 2018), as long as these could still be performed while lying in the MRI scanner. Additional instructions to participants are described in the Supplementary Material.

#### 2.2.5 Real-time fMRI data processing (online analysis)

The fMRI neurofeedback training paradigm was implemented in OpenNFT v1.0 (github.com/OpenNFT/OpenNFT) (Koush et al., 2017). Minimal online pre-processing is described in detail in Koush *et al*. (Koush et al., 2017). It consisted of online motion correction, extraction of the ROIs’ time series, and removal of signal drift, spikes, and high-frequency noise (Koush et al., 2012). Customized scripts were written in MATLAB R2016b using SPM12 to automatically perform visit-wise anatomical (T1) volume reconstruction, coregistration of previous visit’s ROIs, creation of the new functional (EPI) template from current visit’s rs-fMRI data, and initialization of OpenNFT parameters prior to fMRI neurofeedback training. A new functional EPI template was created at each visit from the first 15 rs-fMRI exported volumes, and the previous visit’s template and ROIs were coregistered to the new template for visits 2 to 15. To avoid re-scanning, head motion was monitored in real-time in OpenNFT (Davydov et al., 2022).

#### 2.2.6 Feedback implementation

**Fig. 1:**
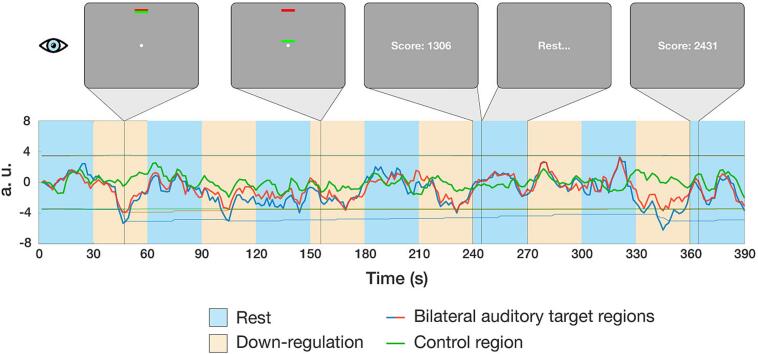
**Continuous visual feedback implementation in a standard fMRI neurofeedback run.** During successful downregulation of bilateral (left: blue; right: red time course) auditory cortices, participants received high feedback on the thermometer bar, as shown during the first trial. When there was no significant downregulation, the feedback bar was closer to the central white dot (e.g., third trial). A cumulative score displayed after each trial provided an indicative performance to the participant. During inter-trial (30 s) resting intervals, the “Rest…” instruction was displayed. (For interpretation of the references to color in this figure legend, the reader is referred to the web version of this article.)

The feedback signal was presented visually on an MRI-compatible screen (BOLD-screen 23 LCD for fMRI, Cambridge Research Systems Ltd., UK) in the form of a simple thermometer moving bar, inspired by previous studies (Caria et al., 2007, Ruiz et al., 2013). It was implemented in Psychophysics Toolbox version 3 (psychtoolbox.org) (Brainard, 1997). The display consisted of a target fixed red bar located at the top of the screen, a white dot at the center, and a moving green regulation bar in between (Fig. 1). Only positive feedback was provided (i.e., the feedback value was kept at zero if participants were to upregulate the average activity of A1). Differential continuous feedback was used (Emmert et al., 2017) to penalize breathing-driven and global BOLD deviation effects on the feedback signal, akin to Sepulveda *et al*. (Sepulveda et al., 2016). The control region was chosen as a larger area involving part of the left motor cortex from a previous pilot finger-tapping motor imagery fMRI neurofeedback experiment (Gninenko, 2022). No clear consensus exists regarding the appropriate choice of such a control region, with an earlier study also using an entire brain slice (Sitaram et al., 2014). Here, the rationale was to select a region a priori not involved in tinnitus, and of larger volume than the targeted auditory ROIs, in order to obtain a smoother global signal estimate and minimize breathing effects on BOLD variability. In addition to the visual feedback, a scoring (reward) system based on bilateral auditory downregulation performance was implemented (Fig. 1), to keep participants motivated throughout the training. Numeric cumulative scores were shown to the participant for ∼ 2 TRs after each regulation block (see Supplementary Material for more details).

#### 2.2.7 Transfer runs without feedback

To assess learning effects with the current fMRI neurofeedback paradigm, transfer runs without continuous feedback were progressively incorporated into the training schedule. Seminal work in the field (deCharms et al., 2004) has shown that some subjects trained through fMRI neurofeedback were able to reproduce similar levels of localized brain activation in the primary somatosensory cortex when feedback was no longer provided. The implementation of transfer runs is further described in the Supplementary Material.

## 3 Data processing and statistical analysis

### 3.1 Pre-processing and analysis of fMRI neurofeedback data (offline analysis)

Functional MRI neurofeedback data were pre-processed using SPM12, with customized code (github.com/ngs5/neurotin) written in MATLAB R2019b. The first and last five volumes from each fMRI neurofeedback run were discarded (as in OpenNFT), yielding 260 volumes per run. All the runs underwent standard pre-processing steps, including slice timing correction, realignment, coregistration to the anatomical (T1) subject-space from the first MRI visit, normalization onto the Montreal Neurological Institute (MNI) space (91 × 109 × 91, 2.0 mm^3^ isotropic) for group analysis, and spatial smoothing with a 6 mm full width at half maximum (FWHM) Gaussian kernel, in subject- or MNI-space. Nuisance regression was performed in subject-space, prior to smoothing, including constant, linear, and quadratic trends, 12 motion (three translational, three rotational, and their first order temporal derivatives), average white matter (WM) and CSF signals, their first order temporal derivatives, and 18 physiological noise (from photoplethysmography and breathing belt recordings) regressors created using the RETROICOR model (Glover et al., 2000) with the PhysIO TAPAS Toolbox (Kasper et al., 2017). All volumes with frame displacement (FD) ≥ 0.5 mm were tagged for fMRI modeling but were not regressed out from the data. A visual quality control was also carried out to exclude data artefacts and ensure proper brain coverage in the pre-processed data, using intersect masks created from all runs per subject.

To assess the effects of group fMRI neurofeedback regulation, first- (run), second- (subject), and third-level (group) general linear modeling (GLM) was performed on the entire data set (1990 runs, including transfer runs). First- and second-level GLMs were modeled in subject-space, with FD regressors included in the first-level model. The third-level model was run with age, gender, and THI at baseline as covariates. Retained bidirectional activation contrasts were inspected at *p* < 0.05 family-wise error corrected (FWE). The corresponding *t*-values were plotted using FSL (fsl.fmrib.ox.ac.uk/fsl) (Jenkinson et al., 2012) with a cluster extent of k = 20. Effect sizes of regulation were reported for several ROIs by computing Cohen’s *d* to account for differences in the total number of runs per subject (Cohen, 2013).

### 3.2 Psychophysiological interaction analysis

Psychophysiological interaction (PPI) (Friston et al., 1997) analyses were performed to extract task-modulated functional connectivity patterns stemming from several seed ROIs, including the bilateral auditory cortex (fMRI neurofeedback target ROIs), bilateral OP3, as well as other seeds of interest revealed by the previous whole-brain analysis. A standard PPI computation approach was performed, with the deconvolution of the seed’s time series before multiplication. Like for the whole-brain analysis, PPI bidirectional contrasts were generated at the threefold level using an additional GLM in SPM12. Connectivity clusters at the group level were then inspected with *p* < 0.05 FWE.

## 4 Results

### 4.1 Participant demographics

A total of 21 participants (49 ± 11.4 years old, 16 males, 5 females) completed the 15 visits of fMRI neurofeedback training. All participants were naive to fMRI neurofeedback prior to the study. On average, participants completed the training in 124.1 ± 48.6 days, totaling 1990 fMRI neurofeedback runs (out of which 391 were transfer runs) with an average of 94.8 ± 6.8 runs per participant. Clinical characteristics of the patients are described elsewhere (Gninenko et al., 2024).

### 4.2 Auditory functional localization

**Fig. 2:**
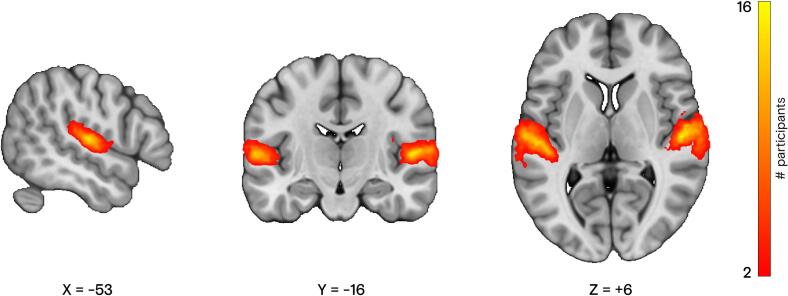
**Auditory functional localization.** Spatial overlap of bilateral auditory localization across the 21 participants who underwent fMRI neurofeedback training. Color bar depicts participant count, with the minimum spatial overlap set to two participants. Coordinates are in MNI space (x: sagittal; y: coronal; z: axial plane). Individual contrasts were initially thresholded at *p*_FWE_ < 0.05.

Bilateral auditory activation was successfully delineated in all participants with contrast thresholds at *p* < 0.05 FWE. Fig. 2 shows the spatial overlap of bilateral auditory activation in MNI space across the 21 participants. Average left and right target ROIs’ sizes were 314.7 ± 63.9 and 321.5 ± 68.5 voxels, respectively, and these did not differ significantly between participants (*p* = 0.41).

### 4.3 Individual and whole-brain group regulation effects

**Fig. 3:**
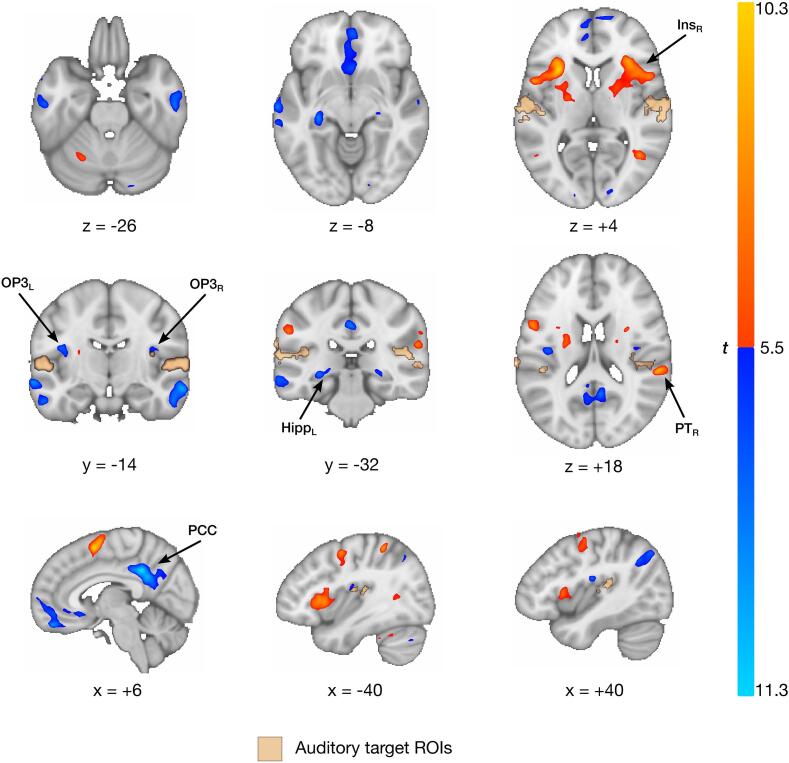
**Whole-brain fMRI neurofeedback regulation outcomes.** Participants upregulated (red; *t* ≥ 5.5) bilateral insula, precentral gyri, right juxtapositional lobule cortex, right superior parietal lobule, left putamen, and a region in the right planum temporale (secondary auditory cortex). Downregulation effects were more pronounced (blue; *t* ≤ -5.5), notably in the posterior cingulate gyrus, bilateral middle temporal gyri, hippocampus, parietal operculum 3, and right superior frontal gyrus. Bilateral auditory neurofeedback target regions are superimposed in beige. Coordinates are in MNI space (x: sagittal, y: coronal; z: axial plane). PCC: posterior cingulate cortex; OP3: parietal operculum 3; PT: planum temporale; Ins: insula; Hipp: hippocampus. (For interpretation of the references to color in this figure legend, the reader is referred to the web version of this article.)

**Fig. 4:**
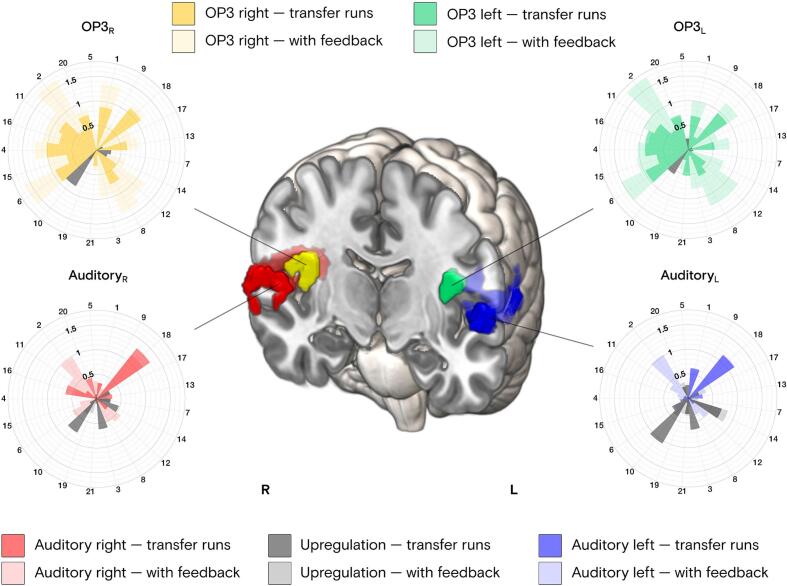
**Individual fMRI neurofeedback downregulation effect sizes (Cohen’s *d*) in bilateral auditory cortex and parietal operculum 3.** Rose plots depict individual downregulation effects (Cohen’s *d*) in each ROI for all the 21 participants. Stronger colors depict effect sizes of runs with continuous visual feedback, and lighter colors depict effect sizes during transfer runs only. Upregulation effect sizes are depicted with the same logic. Functional masks of each ROI are illustrated as whole-brain analysis-derived for bilateral OP3, and as fMRI neurofeedback target ROIs for bilateral auditory cortex.

Group analysis of the 1990 fMRI neurofeedback runs (∼216 h of training) revealed several highly modulated brain regions in the neurofeedback versus rest contrast (Fig. 3, Supplementary Table S1). Strong deactivations were observed in the nodes of the DMN, namely in the precuneus, posterior cingulate cortex (PCC), and medial prefrontal cortex (mPFC), as well as in the bilateral middle (posterior) temporal gyrus, bilateral hippocampus/thalamus, and the right cerebellum. Strong bilateral deactivations in small clusters labelled as OP3, slightly overlapping OP2, were also revealed through careful comparison with the Julich atlas (Amunts et al., 2020). FMRI neurofeedback bilateral auditory target ROIs encompassing parts of A1 and secondary auditory cortex were downregulated, but to a lesser extent, and not by every participant (Fig. 4). Strong activations during fMRI neurofeedback were found in bilateral (mostly anterior) insula, the right juxtapositional lobule cortex, bilateral precentral gyrus, right superior parietal lobule, a cluster overlapping the right middle (temporooccipital part) temporal gyrus, left putamen, and right planum temporale (PT_R_), just posterior to Heschl’s gyrus. No significant activation was found in the control region.

Individual regulation effect sizes (Cohen’s *d*) are depicted in Fig. 4 for bilateral auditory target ROIs and for bilateral OP3, sorted anticlockwise in rose plots according to the fMRI neurofeedback regulation performance (see details in the Supplementary Material). Effect sizes were separately computed for runs with and without continuous visual feedback to assess within-participant learning effects. Cohen’s *d* can be interpreted as ranging from small (*d* = 0.2) to large (*d* > 0.8) effect sizes (Thompson, 2007). The implication of OP3 in tinnitus is further discussed.

### 4.4 Psychophysiological interactions

**Fig. 5:**
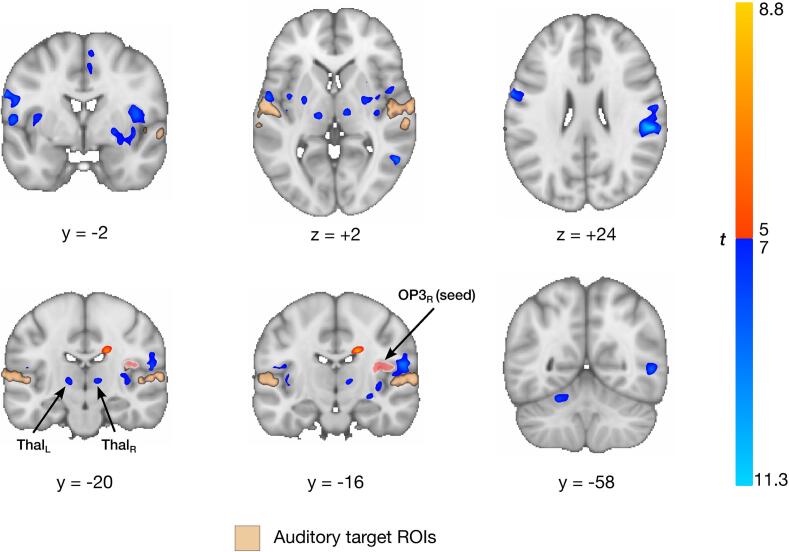
**Task-modulated functional connectivity of right parietal operculum 3 during auditory cortex downregulation.** Regions showing decreased functional connectivity with the right parietal operculum 3 (OP3_R_) during fMRI neurofeedback-mediated downregulation of bilateral auditory cortex. A functional multisensory integration network emerges, involving clusters in the bilateral thalamus, left precentral gyrus, and right juxtapositional lobule cortex, among others (see Table S3).

**Fig. 6:**
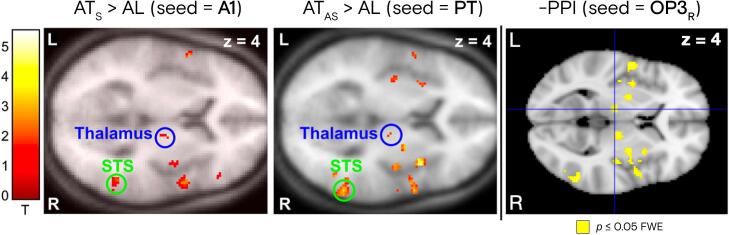
**Parietal operculum 3 desynchronizes from the same multisensory network engaged by auditory cortices during audio-tactile integration.** Left and middle panels reproduce task-modulated functional connectivity networks with A1 and PT as seeds, respectively, reported by Hoefer *et al*. (2013) in the context of a synchronous (AT_S_) and asynchronous (AT_AS_) left bimodal (audio-tactile) stimulation, against acoustic stimulation on the left side only (AL), covaried with subject-specific perceptual sensitivity. The right panel shows negative task-modulated functional connectivity of OP3 in our study during auditory downregulation, highlighting the decrease of connectivity with a very similar functional multisensory integration network during defocalization training from tinnitus through fMRI neurofeedback. Left and middle panels were reproduced with permission from Figure 5 of the original publication (Hoefer et al., 2013).

Task-modulated functional connectivity from several seeds was assessed to highlight potential connectivity relationships between the regulated brain areas. In particular, PPI analyses were conducted seeding from bilateral auditory target ROIs and from bilateral OP3 (as in Job et *al*. (Job et al., 2016, Job et al., 2020); as well as from PT_R_ and bilateral insula (Gninenko, 2022)). These regions were chosen due to their involvement in auditory function, tinnitus, or neurofeedback in general. Seed regions were carefully inspected to exclude possible overlap. Connectivity from bilateral auditory target ROIs revealed six and seven negative interaction clusters when seeding from the left and right ROI, respectively (*p* < 0.05 FWE; Supplementary Table S2). However, only two clusters for the right seed showed no interactions at the single-voxel level, and the decrease in connectivity during neurofeedback appeared to be more pronounced for the left auditory cortex. For the latter, negative interactions were found with clusters in bilateral parietal operculum, bilateral inferior parietal lobule, and contralateral primary auditory cortex. For the right seed, only negative interactions with clusters in ipsilateral parietal operculum (OP1, 3, and 4) were significant. Seeding from left and right OP3 revealed similar but more spatially extended connectivity maps, with mostly negative interactions (Supplementary Table S3). Task-modulated functional connectivity of OP3_R_ is shown in Fig. 5. Negative interactions were mainly found with parts of bilateral OP4, OP1, bilateral thalamus, left cerebellum, bilateral Broca’s area (BA44), bilateral premotor cortex (BA6), and left primary auditory cortex. Similar negative interactions were found when seeding from OP3_L_, with the addition of several clusters, notably one in right amygdala (superficial/centromedial group), suggesting a distributed bilateral network. A more detailed analysis of this network on *Neurosynth* (https://neurosynth.org) revealed a striking similarity with a previous study by Hoefer *et al*. (Hoefer et al., 2013), in which the authors described a very similar network examining the task-modulated functional connectivity of A1 and PT in the context of audio-tactile integration (Fig. 6).

## 5 Discussion

Leveraging this unique data set of 1990 fMRI neurofeedback runs, we identified the brain areas involved in auditory downregulation, thereby demonstrating the involvement of the parietal operculo-insular complex in tinnitus (Job et al., 2016, Jaroszynski et al., 2022). Building on our existing knowledge, this is the longest fMRI neurofeedback study targeting bilateral auditory downregulation in 21 patients with chronic tinnitus over 15 MRI sessions. The results suggest that downregulation of the auditory cortex is accompanied by a decrease in connectivity with OP3, an integration hub also strongly downregulated by most participants. The striking comparison of task-modulated functional connectivity maps stemming from the primary (A1) and secondary (PT) auditory cortices in an unrelated audio-tactile integration task (Hoefer et al., 2013) further reinforces the hypothesis of an altered multisensory integration mechanism in tinnitus, likely mediated by OP3.

### 5.1 Parietal multisensory integration in chronic tinnitus

The involvement of OP3 in tinnitus has previously been hypothesized (Job et al., 2016, Jaroszynski et al., 2022). This brain region has been reported to respond to various suprathreshold nociceptive stimuli, notably demonstrating distinct activation patterns during painful sensations when compared to heat and sound stimuli (Horing et al., 2019). In line with this, prior research has indicated disruptions in somatosensory areas neighboring OP3 among individuals with AAT (Job et al., 2012, Job et al., 2016, Job et al., 2011). Job *et al*. (2011) found that cortical representations of movements related to tympanic membrane pressure were generated bilaterally in focal areas located in the inferior margin of the postcentral gyrus (BA43). Subsequently, authors documented bilateral hyperactivity in adjacent regions BA43/40 among a cohort of 19 individuals with high-pitched tinnitus resulting from AAT, compared to healthy controls, during the execution of an auditory oddball task (Job et al., 2012). Lastly, they observed activity in the right OP3 during the induction of a transient tinnitus percept, which was solely elicited by a specific type of acoustic stimulation (vibrations at 30 Hz, in contrast to 8 Hz, which failed to evoke any transient tinnitus percept) (Job et al., 2016). This acoustic stimulation paradigm was also replicated in a group of tinnitus-free participants undergoing invasive intracortical exploration with stereoelectroencephalography (sEEG) for focal drug-resistant epilepsy. In this group, all (10) but one participant perceived a persisting sound after-effect after acoustic stimulation. Interestingly, the 30 Hz activation was clearly visible on time–frequency plots of all participants with an electrode placed in OP3. These preliminary findings suggest the presence of tonotopic activity in this region, potentially related to either an auditory or somatosensory aspect (specifically, efferent signaling from A1 through dense operculo-temporal connections (Bucheli et al., 2014), or through proprioceptive input via projections from middle ear movements to OP3, respectively (Jaroszynski, 2021)). Additionally, another study conducted with epileptic patients who had undergone implantation of sEEG electrodes for functional mapping reported the emergence of phantom perception descriptors (such as buzzing or hissing), akin to tinnitus, upon invasive stimulation near the posterior OP (Mălîia et al., 2018).

Seminal quantitative cytoarchitectonic analysis enabled the first characterization of four distinct subregions (OP1–4) of the human parietal operculum, highlighting the anatomical correlates behind the heterogeneity of the functionally defined human secondary somatosensory cortex (SII) (Eickhoff et al., 2006, Eickhoff et al., 2006). It was suggested that OP1, OP4, and OP3, constitute the human homologues of primate areas SII, parietal ventral area, and ventral somatosensory area, respectively (Eickhoff et al., 2007). OP1 was shown to be more closely connected to parietal networks, and linked to higher-order somatosensory processing, while OP4 was more closely integrated with areas responsible for action control and basic sensorimotor processing (Eickhoff et al., 2010). OP2, on the other hand, was identified as the human counterpart to the macaque’s parieto-insular vestibular cortex (PIVC) (Eickhoff et al., 2006), displaying cytoarchitectonic characteristics like other primary sensory areas. Subsequent studies have emphasized its potential role as a primary candidate for the human vestibular cortex (Kahane et al., 2003, Lopez et al., 2012, zu Eulenburg et al., 2012, Indovina et al., 2020), although there remains some disagreement on this matter (Devantier et al., 2020), with earlier reports of OP2′s sensitivity to various types of tactile stimulation in humans (Bao et al., 2012). The concept of a convergence region where vestibular and tactile sensations integrate has also been suggested to potentially coexist within OP2 (Beauchamp et al., 2007). Among these areas, the role of OP3 has remained less thoroughly explored. Here, we argue that OP3 may also mediate a form of multisensory integration, notably from auditory and somatosensory (kinesthetic or proprioceptive) crosstalk, and that this integration mechanism is altered in chronic tinnitus, likely leading to phantom perception.

### 5.2 Functional MRI neurofeedback in tinnitus

In this study, deactivations were found in OP3, the bilateral auditory cortex (albeit, to a lesser extent than OP3), the precuneus, PCC, mPFC, as well as in the bilateral middle posterior temporal gyrus, bilateral hippocampus/thalamus, and the right cerebellum during fMRI neurofeedback. Strong activations were found in bilateral anterior insula and PT_R_, just posterior to Heschl’s gyrus. These results are in accordance with the previous findings reported by Haller *et al*. (2010), in which broad deactivations were found in bilateral auditory cortex, in the DMN (PFC, precuneus, and inferior parietal lobe), and increasing activations in bilateral insula extending to the bilateral vlPFC and right dlPFC (Haller et al., 2010). Their study was the first to examine the hypothesis that individuals experiencing tinnitus could learn to volitionally decrease heightened activity in the auditory cortex through fMRI neurofeedback, and whether this process would result in a reduction of tinnitus symptoms. They reported that a small sample of five out of six chronic tinnitus sufferers successfully downregulated broad auditory cortex activity over four short fMRI neurofeedback sessions of 4 min 24 s each, and two participants reported a mild improvement in tinnitus symptoms after the training.

The DMN, which is known to deactivate upon cognitive load (Raichle et al., 2001), and its subnetworks, were shown to recover in an anterior-to-posterior fashion after demanding downregulation of the primary auditory cortex in healthy subjects, with the level of task engagement inversely influencing the recovery of brain regions related to attention compared to those related to internally directed cognition (Van De Ville et al., 2012). Furthermore, downregulation of the auditory cortex through fMRI neurofeedback resulted in increased connectivity with subcortical regions of the brainstem, the insula, and low- and high-level visual, attention, and working-memory networks (Haller et al., 2013). In a subsequent study, authors showed that continuously presented visual feedback was more advantageous than its intermittent alternative for longer-term fMRI neurofeedback training of auditory cortex downregulation (Emmert et al., 2017). Moreover, the downregulation effect was more pronounced in the secondary auditory cortex. Another research group showed that healthy participants who received a genuine feedback signal displayed a notable reduction in auditory cortex activity compared to those receiving yoked feedback (Sherwood et al., 2019). This decrease was observed in response to continuous acoustic noise stimulation during fMRI neurofeedback training. Additionally, significant correlations were found between changes in volitional control over auditory cortex activity and subjective assessments of attentional control. Collectively, these findings brought the fMRI neurofeedback methodology a step closer to being clinically relevant, directly showcasing its clinical potential in comparison to CBT for individuals dealing with chronic tinnitus, with clinical benefits persisting for up to 12 months (Gninenko et al., 2024).

The insula is known to be implicated in interoceptive awareness and cognitive control (Critchley et al., 2004), in allocating auditory attention, and in visual-auditory integration (Bamiou et al., 2003). Its activation in fMRI neurofeedback studies has consistently been reported (Emmert et al., 2016). The anterior insula (aI), in conjunction with the dorsal anterior cingulate cortex (dACC), constitute the majority of the brain’s salience network, which filters the most relevant of all available internal and external stimuli. In chronic tinnitus, increased activation of both areas has been reported (Elgoyhen et al., 2015) and proposed to be associated with the salience assigned to the phantom percept, potentially impeding habituation (De Ridder et al., 2014, De Ridder et al., 2011). The robust bilateral activation of aI observed in our study aligns with existing evidence from fMRI neurofeedback (Sitaram et al., 2017). The latter has been attributed to a part of the reward processing network in the brain and is likely to supersede any potential hyperactivity that could be attributed to tinnitus, making it challenging to discern a distinct interpretation from the current findings.

Sustained activation in the vicinity of PT_R_ has previously not been documented in fMRI neurofeedback literature (Sitaram et al., 2017). This region raises interest related to the pathophysiology of chronic tinnitus. PT is a highly asymmetric brain region located posterior to Heschl’s gyrus within the Sylvian fissure, considered part of the secondary auditory cortex. Beyond its involvement in language processing, this area has been suggested to contribute to an auditory attentional network, although its computational function is not contingent upon attention (Griffiths and Warren, 2002), but rather linked to the processing of spectrally and temporally complex sounds. A prior PET study has associated the experience of gaze-evoked tinnitus with increased activity in bilateral areas near the junction of PT and planum parietale (Giraud et al., 1999). However, the reported hyperactivity was more pronounced in the left hemisphere. In our study, the consistent activation observed in right PT could be related to the ongoing (sub)conscious reassessment of the phantom percept during cognitively demanding defocalization strategies that entail shifting attention during fMRI neurofeedback downregulation trials.

Collectively, these findings indicate that OP3 plays a key role in integrating multiple sensory modalities, including somatosensory (potentially involving middle ear proprioception (Job et al., 2016)) and auditory information. In chronic tinnitus, the integration of multiple sensory inputs appears to be disrupted (Jaroszynski et al., 2022), possibly due to both mechanical damage to the middle ear transmitted via the somatosensory pathway and noise-induced damage transmitted via the auditory pathway, as exemplified by AAT. In our study, participants decreased their neuronal activity in both bilateral OP3 and (to a lesser degree) bilateral auditory cortex, while simultaneously diminishing FC between these regions during downregulation trials, presumably through a mechanism of learned modulation (Shibata, 2021). Moreover, we have demonstrated that OP3 decreased its FC with the same multisensory integration network as the one recruited by A1 and PT during an audio-tactile interplay task (Hoefer et al., 2013). Remarkably, within this investigation, the strength of task-modulated functional connectivity was found to have a direct correlation with the subjects’ perceptual sensitivity. This finding aligns with the hypothesis that participants in our study were able to alleviate their perceptual sensitivity to tinnitus by diminishing the FC of OP3 with this network during fMRI neurofeedback, subsequently leading to a substantial reduction of tinnitus burden (Gninenko et al., 2024).

This clinical trial aimed to investigate the superiority of neurofeedback over CBT for tinnitus relief. The fMRI neurofeedback arm discussed here suffers from a few limitations, such as its technically challenging aspects and elevated cost, the possibility to only include a control region for feedback computation, and the lack of a proper within-arm control group (e.g., with inverted or sham feedback), due to the length of the training, and to the ethically unreasonable prospect of providing inverted feedback (i.e., auditory upregulation) to highly suffering tinnitus patients. The development of alternatives, such as fNIRS neurofeedback protocols, could enhance translation into clinics. Nevertheless, our neuroimaging findings tend to confirm the hypothesis of OP3′s involvement (Job et al., 2016), and support its important role as a hub mediating altered multisensory integration in chronic tinnitus. Future work could include behavioral multisensory assessments to better delineate the observed effects. Current promising bimodal neuromodulation techniques that indirectly target the trigeminal nerve, combined with acoustic or electrical modulation of the auditory pathway, could benefit from future combined neuroimaging studies to probe this mechanism, thereby also accelerating the increase of treatment efficacy.

## CRediT authorship contribution statement

**Nicolas Gninenko:** Writing – review & editing, Writing – original draft, Visualization, Software, Project administration, Methodology, Investigation, Formal analysis, Data curation, Conceptualization. **Pascal Senn:** Writing – review & editing, Project administration, Funding acquisition, Conceptualization. **Sven Haller:** Writing – review & editing, Resources, Conceptualization. **Dimitri Van De Ville:** Writing – review & editing, Supervision, Resources, Project administration, Funding acquisition, Conceptualization.

## Funding

This research was, in major part, funded by the Wyss Center for Bio and Neuroengineering (Geneva, Switzerland).

Competing interests.

NG reports a financial relationship with Neurosoft Bioelectronics, a company pursuing novel tinnitus treatment development. This relationship is unrelated to the present work, which was completed independently and without any involvement or influence from the company.

## Declaration of competing interest

The authors declare that they have no known competing financial interests or personal relationships that could have appeared to influence the work reported in this paper.

## Data Availability

The data that support the findings of this study are available upon reasonable request from the corresponding author. MRI data are not publicly available due to their containing information that could compromise the privacy of research participants.
