# Supplementary material for: Altered parietal multisensory integration in chronic tinnitus during closed-loop real-time fMRI auditory downregulation
Source: Neuroimage Clin. 2026 Feb 12;49:103960. doi: 10.1016/j.nicl.2026.103960 (PMC12930045; doi:10.1016/j.nicl.2026.103960)
Supplement: Supplementary Data 1 [file mmc1.docx]

**Supplementary Material to**

Altered parietal multisensory integration in chronic tinnitus
during closed-loop real-time fMRI auditory downregulation

Nicolas Gninenko, Pascal Senn, Sven Haller and Dimitri Van De Ville

**Study design**

We have included, at the end of this document, the CRED-NF checklist^1^ — the consensus guidelines for reporting and designing clinical and cognitive-behavioral neurofeedback studies — along with explanations and references relevant to our study design.

**Additional data acquisition details**

Deformation (b_0_) field maps were acquired with TR = 627 ms, anterior to posterior phase encoding (A≫P), TE_1_ = 5.19 ms, TE_2_ = 7.65 ms, 106×106×64 resolution with no gap, FA = 60^◦^, 2.0 mm^3^ isotropic voxel size, 192 volumes (∼3 min). Diffusion-weighted imaging (DWI) was also acquired at visits 1 and 15, with an interleaved multislice sequence (accel. factor = 6) with 30 directions, b_0_ = 1000 s/mm^2^, TR = 4500 ms, A≫P, TE = 60 ms, 150×150×96 without gap, 1.5 mm^3^ isotropic voxel size (∼3.5 min). Physiological data (photoplethysmography and respiration belt) were acquired during all functional sequences using BIOPAC MP150 (RSP100C amplifier, BIOPAC Systems Inc., Goleta, USA) with the AcqKnowledge 4.4.1 software for offline pre-processing. Participants were instructed not to think of anything and to close their eyes during resting-state fMRI, which was monitored outside the scanner with an EyeLink 1000+ eye tracker (SR Research, Canada). Acquisition parameters (in-plane resolution, number of z-slices, TR) were optimized during pilot recordings^2^ according to the data export and processing capabilities of the real-time computer at the MRI facility, to ensure that no lag would accumulate during continuous visual feedback presentation, balanced with an acceptable coverage of the brain. The real-time fMRI neurofeedback setup ran on a Dell Precision Tower 5810, Intel Xeon E5-1650 v3, 3.5 GHz, 32 Gb RAM, with a NVIDIA Quadro K5200 (8 Gb RAM) on Windows 7.

**Detailed instructions to participants**

Personalized guidance for cognitive strategies during neurofeedback was provided through brief debriefing interviews after each session. Participants' employed strategies were documented, and their effectiveness and suitability for downregulation were discussed. Upon request, participants could be reminded of their most effective strategies at any time. If participants struggled to explore new cognitive approaches after several sessions, general, non-specific cues (e.g., “positive memories,” “emotion”) were orally suggested as prompts.

For visual feedback, participants were instructed to keep the regulation bar — displayed on the MR-compatible screen — as high and as sustained as possible. They were informed about the purpose of the study and the intrinsic 4–5 second delay of the blood-oxygen-level-dependent (BOLD) response, without revealing the precise feedback computation mechanism. Participants were also instructed to avoid body, limb, and head movement during scans, which was monitored using the built-in MRI safety camera. Additionally, they were asked to refrain from consuming caffeinated or alcoholic beverages prior to each visit.

**Details on the computation of the visual feedback**

To reduce the influence of breathing-related and global BOLD signal fluctuations on the feedback, a differential continuous feedback signal was computed, similar to the method described by Sepulveda *et al*.^3^ The control region was derived from prior finger-tapping motor imagery neurofeedback pilot experiments^2^ and included a portion of the left motor cortex.

Feedback estimation for each ROI was done as follows: the median signal at time *t* was computed over the window [*t* − 2, *t*], and the median baseline from the preceding 30-second rest block (excluding [0–3 s] and the final 2 s to correct for anticipation and habituation effects) was subtracted. The mean of both auditory ROIs was computed, from which the control ROI signal was subtracted to yield the final feedback value. This signal was then inversely scaled to the [0, 100] range for visual display, with higher values reflecting stronger bilateral auditory cortex downregulation.

Participants were instructed to avoid any motor strategies and remained under continuous video monitoring. Hand movement was further restricted by having one hand hold the MRI emergency squeeze ball and the other fitted with a photoplethysmography device.

In addition to the continuous visual feedback (the green regulation bar updated every TR), a performance scoring system was implemented. After each regulation block, participants were shown a numeric score (lasting for ~2 TRs), calculated as the cumulative sum of the feedback bar value during that preceding block. Scores ranged from 1 (minimal overlap between the green bar and the central white dot) to 100 (complete overlap with the red target bar). Although participants were informed of the cumulative nature of the score, the exact algorithm was not disclosed to prevent bias.

This scoring system served a motivational purpose across the 15-session protocol. Scores were discussed at the end of each visit to maintain engagement, acting as a non-monetary reward and a form of intermittent feedback complementing the continuous regulation bar. This design also helped alleviate monotony associated with the repetitive nature of extended neurofeedback training.

**Transfer runs without feedback**

This study incorporated both “transfer” and “no-feedback” runs, in which the green regulation bar and red target bar were removed from the screen during regulation blocks (see **Figure 1** in the main text for standard visual feedback). During transfer runs, participants viewed only a central dot during regulation but still received a score after each block. This minimal form of intermittent feedback still allowed participants to self-assess the effectiveness of their cognitive strategies across trials. However, since the scoring algorithm was undisclosed, the feedback was relative — participants could compare scores across runs but had no reference for the magnitude of downregulation achieved.

In contrast, no-feedback runs offered no performance information at all; participants remained entirely unaware of their regulation outcomes. Two transfer runs were included at visits 5, 7, 8, and 10–15, while two no-feedback runs were introduced only at visits 14 and 15 (see Figure S2 in Gninenko *et al*.^4^).

**Results**

**Functional MRI neurofeedback regulation performance**

To supplement raw neurofeedback scores with statistical significance, we aimed to estimate a proper chance level for auditory downregulation within each neurofeedback run. One approach — using session-specific rs-fMRI data to compute reference scores based on the three target ROIs (bilateral auditory and control) — was discarded. While feasible, this would have confounded comparisons across runs within the same session due to intra-session variability in baseline signals, especially in the context of potential learning effects.

Instead, we adopted a surrogate data testing approach based on the neurofeedback data itself. This method enables null-hypothesis testing for time-series nonlinearity.^5,6^ Specifically, we applied phase randomization to ROI time courses using the Amplitude Adjusted Fourier Transform (AAFT), which preserves both amplitude distribution and linear correlations. Although the AAFT includes a Gaussianization and de-Gaussianization step that may slightly distort the linear structure, it better preserved amplitude fluctuations across ROIs — an essential feature for a nonlinear scoring metric.

We ensured consistent phase randomization across the three ROIs, following recommendations in the field.^7^ Inconsistent randomization would violate the null hypothesis of linear, stationary Gaussian processes, and indeed yielded overly wide surrogate distributions that precluded significance, even in strong neurofeedback runs.^8,9^ We therefore implemented a consistent AAFT procedure^10^ (adapted from a MathWorks File Exchange script^[[1]](#footnote-1)^) tailored to our dataset.

From each neurofeedback run, we extracted processed time courses for the three ROIs (saved via OpenNFT) and generated 1,000 surrogate triplets per run. Surrogate scores were computed using the same feedback formula and aggregated into run-specific distributions. A run’s real score was considered significant if it ranked in the top 5% of its surrogate distribution (i.e., rank ≤ 50, *p* < 0.05). Across participants, we then calculated the proportion of significant runs and modelled this using a binomial distribution to assess the probability of the observed outcomes.

Participants were then ranked anticlockwise according to neurofeedback downregulation performance (see **Figure 4** in the main text). To account for slight variations in the total number of neurofeedback runs across participants, corresponding downregulation *t*-values for each ROI and each participant were converted to Cohen’s *d* effect sizes. Effect sizes were computed separately for runs with and without continuous visual feedback to assess within-participant learning effects. Transfer runs were grouped with the few no-feedback runs for this analysis.

**Supplementary Tables**


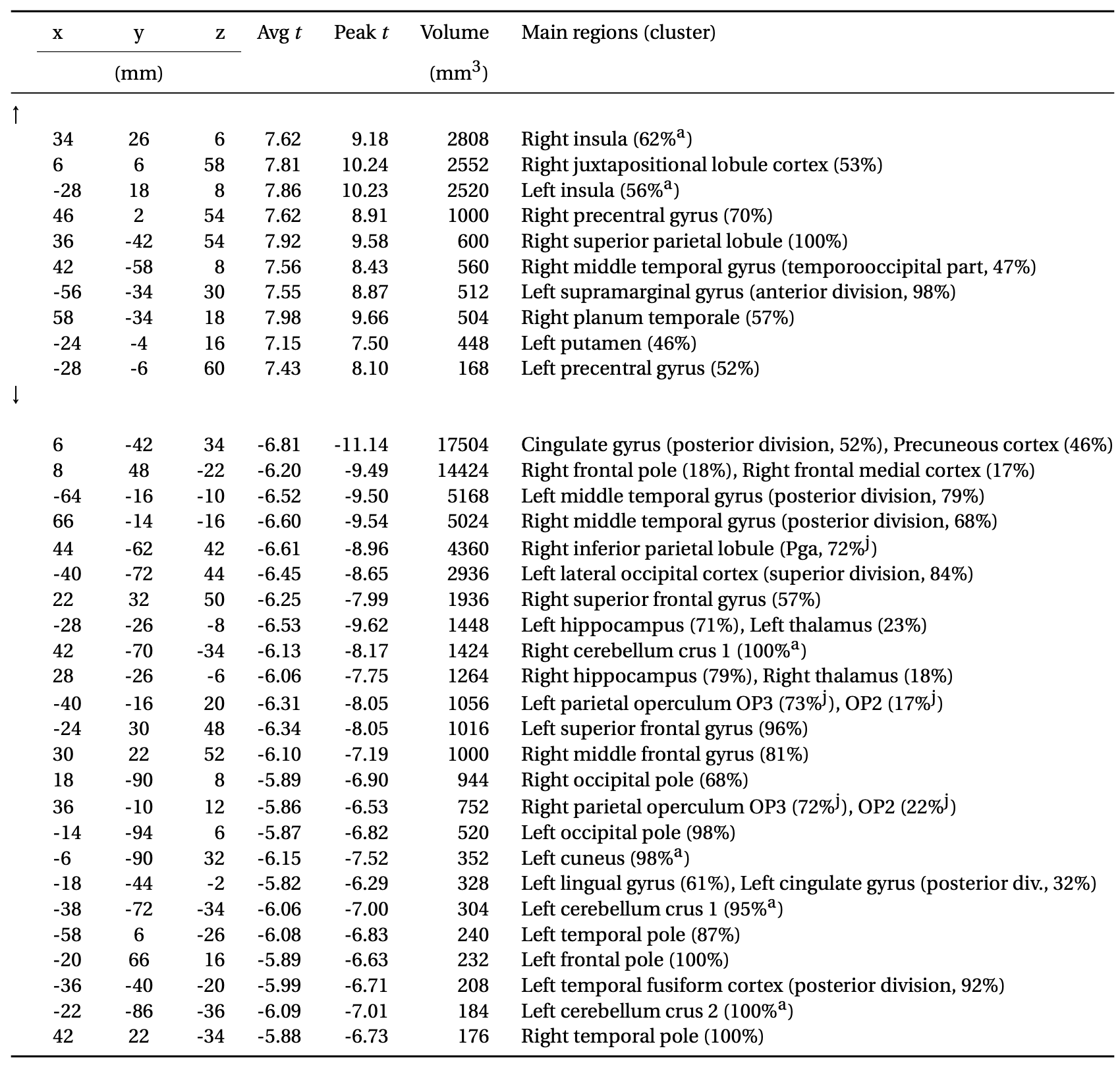


**Table S1** — Functional MRI neurofeedback whole-brain group regulation correlates from 1990 runs from 21 participants (|*t*| > 5.5, cluster extent *k* = 20). MNI-coordinates (x, y, z in mm), average *t*-value in clusters, peak *t*-value, volume (in mm^3^), and most probable anatomical locations (at cluster level) according to the Harvard-Oxford atlas^11–14^ (or **^a^**: AAL atlas^15^, or **^j^**: Julich atlas^16^, when mentioned otherwise) are reported. ↑: activations, ↓: deactivations.


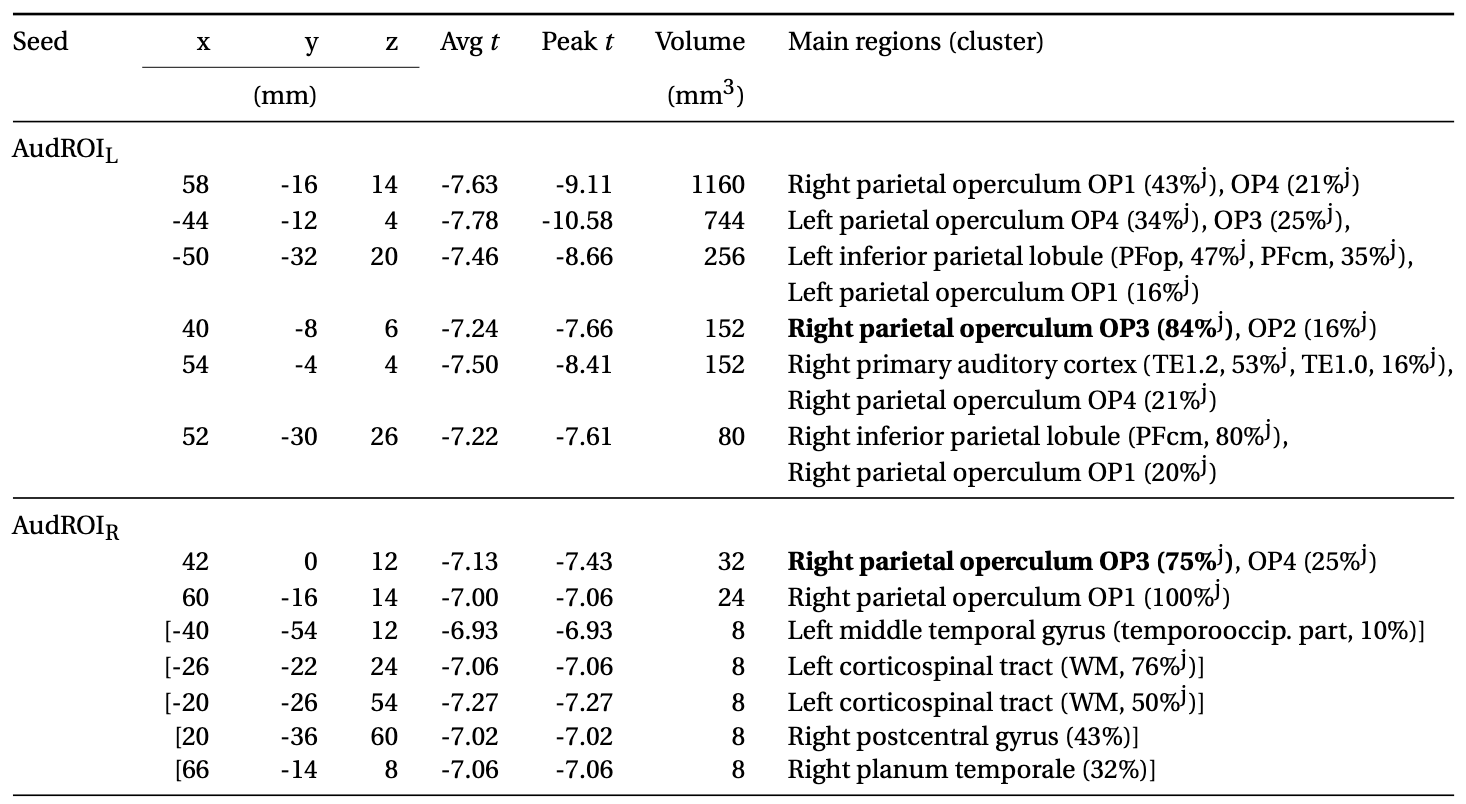


**Table S2** — Negative psychophysiological interactions (PPI) for bilateral auditory target ROIs (AudROI_L_: left; AudROI_R_: right; 21 participants, 1990 fMRI neurofeedback runs, *p* < 0.05 FWE, cluster extent *k* = 10). MNI-coordinates (x, y, z in mm), average *t*-value in clusters, peak *t*-value, volume (in mm^3^), and most probable anatomical locations (at cluster level) according to the Harvard-Oxford atlas^11–14^ (or **^a^**: AAL atlas^15^, or **^j^**: Julich atlas^16^, when mentioned otherwise) are reported. For AudROI_R_, 5 single-voxel clusters (marked between [brackets]) are included for completeness (when setting *k* to 0). No significant positive interactions were found.


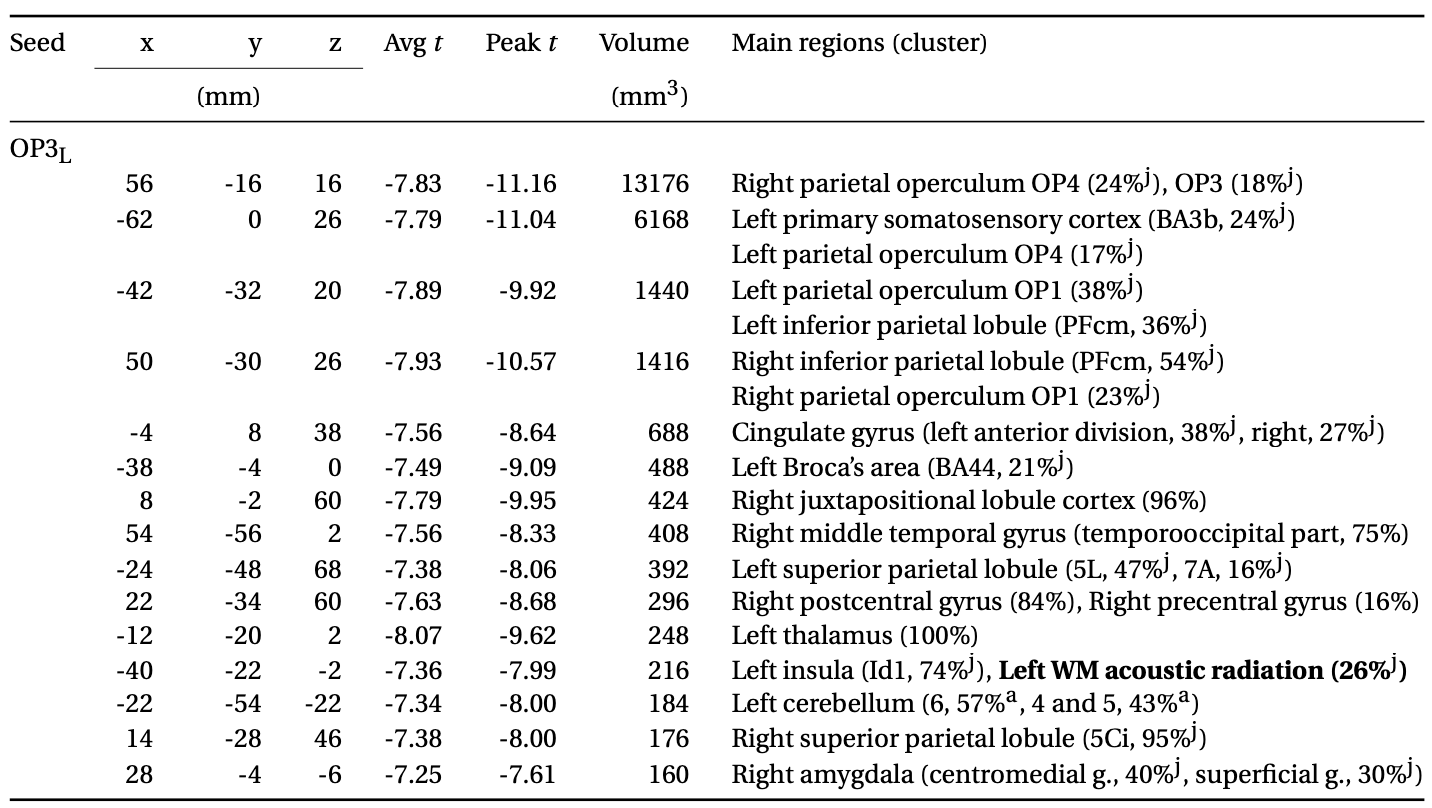


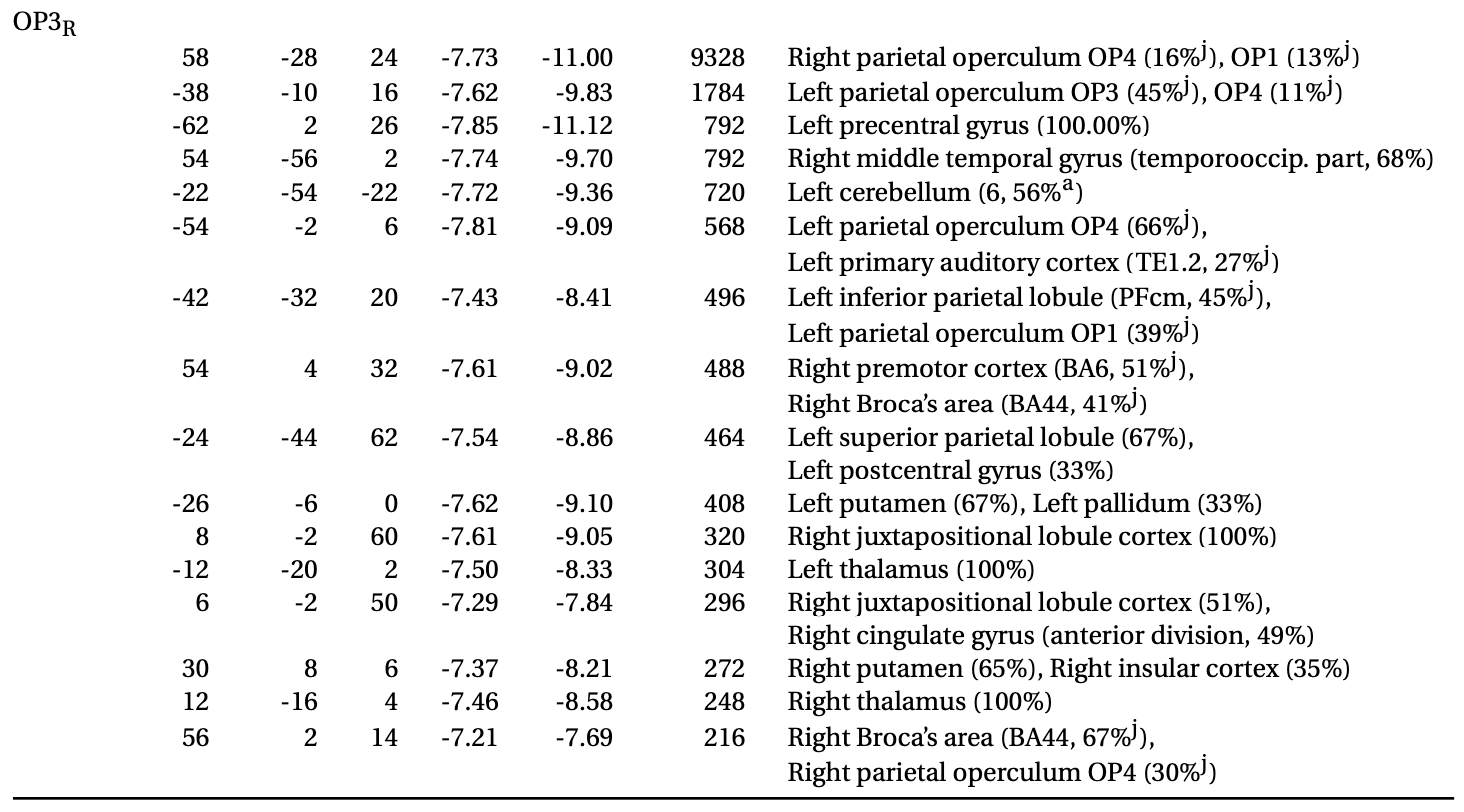


**Table S3** — Negative psychophysiological interactions (PPI) for bilateral parietal operculum 3 (OP3; 21 participants, 1990 fMRI neurofeedback runs, *p* < 0.05 FWE, cluster extent *k* = 20). MNI-coordinates (x, y, z in mm), average *t*-value in clusters, peak *t*-value, volume (in mm^3^), and most probable anatomical locations (at cluster level) according to the Harvard-Oxford atlas^11–14^ (or **^a^**: AAL atlas^15^, or **^j^**: Julich atlas^16^, when mentioned otherwise) are reported. No significant positive interactions were found. BA: Brodmann area, g.: group, temporooccip.: temporooccipital. Only one positive interaction remained significant (see also Figure 5 in the main text): it was localized within the right superior occipitofrontal fascicle in white matter (54%, as per Julich atlas^16^) at MNI peak coordinates [22 -16 30].

**References (Supplementary Material)**

1. Ros T, Enriquez-Geppert S, Zotev V, et al. Consensus on the reporting and experimental design of clinical and cognitive-behavioural neurofeedback studies (CRED-nf checklist). *Brain*. 2020;143(6):1674-1685. doi:10.1093/brain/awaa009

2. Gninenko N. *Real-Time fMRI Neurofeedback: Methods and Application to Chronic Tinnitus*. EPFL; 2022. doi:10.5075/epfl-thesis-8774

3. Sepulveda P, Sitaram R, Rana M, Montalba C, Tejos C, Ruiz S. How feedback, motor imagery, and reward influence brain self-regulation using real-time fMRI. *Human Brain Mapping*. 2016;37(9):3153-3171. doi:10.1002/hbm.23228

4. Gninenko N, Trznadel S, Daskalou D, et al. Functional MRI Neurofeedback Outperforms Cognitive Behavioral Therapy for Reducing Tinnitus Distress: A Prospective Randomized Clinical Trial. *Radiology*. 2024;310(2):e231143. doi:10.1148/radiol.231143

5. Theiler J, Eubank S, Longtin A, Galdrikian B, Doyne Farmer J. Testing for nonlinearity in time series: the method of surrogate data. *Physica D: Nonlinear Phenomena*. 1992;58(1):77-94. doi:10.1016/0167-2789(92)90102-S

6. Prichard D, Theiler J. Generating surrogate data for time series with several simultaneously measured variables. *Phys Rev Lett*. 1994;73(7):951-954. doi:10.1103/PhysRevLett.73.951

7. Abrol A, Damaraju E, Miller RL, et al. Replicability of time-varying connectivity patterns in large resting state fMRI samples. *NeuroImage*. 2017;163:160-176. doi:10.1016/j.neuroimage.2017.09.020

8. Borgnat P, Flandrin P, Honeine P, Richard C, Xiao J. Testing Stationarity With Surrogates: A Time-Frequency Approach. *IEEE Transactions on Signal Processing*. 2010;58(7):3459-3470. doi:10.1109/TSP.2010.2043971

9. Liégeois R, Laumann TO, Snyder AZ, Zhou J, Yeo BTT. Interpreting temporal fluctuations in resting-state functional connectivity MRI. *NeuroImage*. 2017;163:437-455. doi:10.1016/j.neuroimage.2017.09.012

10. Kugiumtzis D. Surrogate data test for nonlinearity including nonmonotonic transforms. *Phys Rev E*. 2000;62(1):R25-R28. doi:10.1103/PhysRevE.62.R25

11. Makris N, Goldstein JM, Kennedy D, et al. Decreased volume of left and total anterior insular lobule in schizophrenia. *Schizophrenia Research*. 2006;83(2):155-171. doi:10.1016/j.schres.2005.11.020

12. Frazier JA, Chiu S, Breeze JL, et al. Structural Brain Magnetic Resonance Imaging of Limbic and Thalamic Volumes in Pediatric Bipolar Disorder. *AJP*. 2005;162(7):1256-1265. doi:10.1176/appi.ajp.162.7.1256

13. Desikan RS, Ségonne F, Fischl B, et al. An automated labeling system for subdividing the human cerebral cortex on MRI scans into gyral based regions of interest. *NeuroImage*. 2006;31(3):968-980. doi:10.1016/j.neuroimage.2006.01.021

14. Goldstein JM, Seidman LJ, Makris N, et al. Hypothalamic Abnormalities in Schizophrenia: Sex Effects and Genetic Vulnerability. *Biological Psychiatry*. 2007;61(8):935-945. doi:10.1016/j.biopsych.2006.06.027

15. Tzourio-Mazoyer N, Landeau B, Papathanassiou D, et al. Automated Anatomical Labeling of Activations in SPM Using a Macroscopic Anatomical Parcellation of the MNI MRI Single-Subject Brain. *NeuroImage*. 2002;15(1):273-289. doi:10.1006/nimg.2001.0978

16. Amunts K, Mohlberg H, Bludau S, Zilles K. Julich-Brain: A 3D probabilistic atlas of the human brain’s cytoarchitecture. *Science*. Published online August 21, 2020. Accessed January 17, 2022. https://www.science.org/doi/abs/10.1126/science.abb4588

1. <https://ch.mathworks.com/matlabcentral/fileexchange/16062-test-of-non-linearity>. [↑](#footnote-ref-1)
